# Violence against Women in a Slum Area in Helwan, Cairo, Egypt: A Community Based Survey

**DOI:** 10.34172/jrhs.2020.01

**Published:** 2020-01-21

**Authors:** Nessrin Ahmed El-Nimr, Salma Mohammed Gouda, Iman Mohamed Helmy Wahdan

**Affiliations:** ^1^Department of Epidemiology, High Institute of Public Health, Alexandria University, Egypt; ^2^FGM joint program coordinator, UNFPA Arab States Regional Office, Cairo, Egypt

**Keywords:** Egypt, Gender-based violence, Intimate partner violence, Prevalence

## Abstract

**Background:** Violence against women (VAW) is a major global public health problem with serious consequences. We aimed to estimate the prevalence of VAW aged 18-45 yr in a slum area in Helwan, Cairo, to assess their knowledge and perspective regarding VAW, and to assess their help-seeking practice in response to violence.

**Study design:** Cross-sectional design.

**Methods:** This community based survey was carried out among 657 women in a slum area in Helwan, Cairo, Egypt in 2018. Data about the women’s knowledge about VAW, exposure to different forms of violence and their frequency, women’s perspective towards violence, and their healthcare-seeking behavior on exposure to violence were collected using an interviewing questionnaire.

**Results:** The prevalence of exposure to at least one type of intimate partner violence (IPV) was 59.1% with psychological violence ranking 1^st^ followed by physical violence. Most women exposed to IPV reported that they have never asked for healthcare upon exposure to violence. One third had good knowledge. Most had favorable perspective against VAW.

**Conclusion:** Most women suffered some kind of violence. They, however, did not seek help most of the time.

## Introduction

According toWHO, violence was defines as "the intentional use of physical force or power, threatened or actual, against oneself, another person, or against a group or community that either result in or has a high likelihood of resulting in injury, death, psychological harm, maldevelopment or deprivation". Violence according to gender of the person on whom the violent act is implicated is called gender-based violence (GBV) ^1^.

One of the major public health problems is violence against women (VAW) considered a contravention of human rights. Its most common type is intimate partner violence (IPV) ^2^. The United Nations defined VAW as "any act of GBV that results in or is likely to result in, physical, sexual or mental harm or suffering to women, including threats of such acts, coercion or arbitrary deprivation of liberty, whether occurring in public or in private life" ^3^. About 30% of women who have been in a relationship have experienced IPV, namely physical and/or sexual. In the Eastern Mediterranean region, levels were as high as 37% ^2^.

In 2013, about 42% of women experienced IPV reported an injury as a consequence of that violence. Victims of violence were also at double the risk of contracting suicide, substance use problems, a 1.5-fold rise in the risk of having sexually transmitted infections, and a 16% increase in the risk of having a low birth weight infant ^4^.

Women are more likely to experience violence in the event that they had low level of education, had mothers being abused by way of a partner, had been abused for the duration of childhood, or had attitudes accepting violence. Other risk factors for experiencing violence included community norms of male privilege or women’s subordinate status and low levels of women’s access to paid employment ^2^.

In Egypt, about 30% of ever-married women aged 15-49 yr have ever experienced some form of spousal violence. About 25% were subjected to physical violence, 19% to emotional violence, and 4% to sexual violence. Only one-third of women who experienced violence since the age of 15 yr ever sought help to deal with violence ^5^. Most women who did not seek help thought that violence is a part of life or was ashamed of being abused ^6^.

Obtaining reliable data on the extent of violence, its causes and consequences is important for the development of local action plans as well as preventive programs and services. Many cases of VAW do not come to the attention of the authorities or service providers, rendering community-based studies a vital source of information to track the nature and extent of these issues^7^. Similar recommendations were reported in Alexandria, Egypt in addition to mobilizing public efforts against VAW and enhancing knowledge about gender roles in Egypt ^8^.

The aim of the study was to estimate the prevalence of VAW aged 18-45 yr in a slum area in Helwan, Cairo, Egypt, to assess their knowledge, perspective and healthcare-seeking practice in response to VAW.

## Methods

A community-based household survey was carried out from Mar until May 2018 among adult women aged 18-45 yr living in Helwan, Cairo Governorate, Egypt.

The study protocol was reviewed and approved by the Ethics Committee of the High Institute of Public Health, Alexandria University. The researchers complied with the International Guidelines for Research Ethics. Informed verbal consent was obtained from all participants after explanation of the purposes and benefits of the research. Anonymity and confidentiality of information were assured and maintained. All participants had the right to participate and to withdraw from the study once it has started. Respect of all participants was ensured. The quality and integrity of research were ensured. There was no conflict of interest. All researchers cooperated and participated in all phases of the research, reviewed all research results, analyses and interpretations. They were all involved in decision making about publication.

Helwan is a part of Grater Cairo, on the bank of the river Nile. It consists of seven geographical districts. Governmental health services in Helwan include three hospitals, two maternal and child health centers, seven separate health offices, seven health centers (containing health offices), five health centers (with no health offices) and one clinic.

The sample size was calculated using Epi Info version 7.2.0.1, 2016. Based on a prevalence of violence against women of 30% ^5^, 5% confidence limit, and design effect of 2, the minimum required sample size at 95% confidence level was calculated to be 646 women. A representative sample of households was drawn from the eight geographic districts of Helwan, taking into consideration the total size of the population in each region. Thirty clusters were selected representing the different zones of each region using probability proportionate to size. Each cluster was composed of 30 households and all adult women aged 18-45 yr who were present at the time of the interview were included. The sample amounted to 750 women, but complete information were possible to obtain from 657 women (response rate=87.6%).

Data were collected using a predesigned structured interviewing questionnaire. The questionnaire was prepared guided by the Centers for Disease Control and Prevention (CDC) assessment tools measuring violence-related attitudes, behaviors and influences among youth ^9^ and the assessment questionnaire used in the Egyptian Health Demographic survey 2014 ^5^.

The questionnaire consisted of five sections: The first included personal and demographic data (age, education, residence, occupation and accessibility of healthcare services). The second was concerned with the women’s knowledge about VAW. It consisted of 10 single and multiple response questions about the meaning of violence, gender and VAW. The participants were asked to identify violence from a list of different behaviors/situations, and identifying some physical and mental symptoms as effects of violence. A scoring system was constructed and the total knowledge score ranged from 0 to 22, classified into three levels: good (≥75% or ≥17 points), fair (50% to <75% or 11 to <17 points) and poor (<50% or 0 to <11 points).

The third section included 26 questions about the exposure to different forms of violence (physical, psychological or sexual) during the previous 12 months, their frequency and the relation with the perpetrator(s) (intimate or non-intimate partner). The fourth section was for the women’s perspective towards violence and included 13 statements or situations in the context of VAW. For each statement, women were asked to identify (on a five-point Likert scale) the extent to which they agreed or disagreed with the given situations picturing personal beliefs, cultural norms and social consequences of violent behaviors. The total perspective score ranged from 13 to 65 and was classified into favorable (≥75%), fair (50% to <75%) and unfavorable (<50%) levels of perspective. The fifth section was for assessment of the healthcare-seeking behavior on exposure to violence. It included ten questions about whether women asked for healthcare after being exposed to violence and its frequency, reasons for seeking and for not seeking healthcare and the places where healthcare was sought.

Discriminant validity was used to validate the knowledge and attitude scales by discriminating between the scores of the first quartile (≤Q1) and the third quartile (≥Q3) using the Mann-Whitney Test. The first and third quartiles of the knowledge score were statistically significant (Z= -16.24, *P* =0.010; Median (IQR) were 10 (4) and 19 (3), respectively). Similarly, the first and third quartiles of the attitude score were statistically significant (Z= -16.29, *P* =0.010; Median (IQR) were 44 (7) and 59 (3), respectively). The internal consistency was tested and Cronbach’s alpha=0.768.

The collected data were revised, coded and fed to statistical software IBM using SPSS for Windows version 21.0 (SPSS Inc., Chicago, IL, USA). For quantitative variables, mean and standard deviation were calculated. Using three stepwise logistic regression models, the magnitude of the association between different variables and the level of knowledge and perspectives of women regarding VAW and the exposure of ever-married women to VAW was estimated.All statistical analyses were done using two-tailed tests and *P* -value less than 0.05 was considered to be statistically significant.

## Results

The mean age of women was 32 ±7.7 yr with a median of 33 yr. Nearly half (47.2%) were in the age group of 30 to <40 yr, 71.3% were married, 22.1% were illiterates or could just read and write and 75.0% were housewives.

Among the 524 ever-married women, 514 (98.1%) agreed to answer the question about exposure to IPV. All the 514 (98.1%) agreed to answer questions about psychological and physical violence, while 496 (94.7%) agreed to answer questions about exposure to sexual violence. The prevalence of exposure to at least one type of IPV was 59.1% (304). Exposure to psychological and physical violence from an intimate partner was reported by 49.1% (252) and 37.3% (192) of women, respectively, while exposure to sexual violence by an intimate partner was reported by only 6.4% (32).

Regarding psychological violence, about one-third of women reported being humiliated by her intimate partner, or being called names, while 7.2% reported being threatened to be harmed (Table 1). As regards physical violence, about one in every four reported being pushed by her intimate partner, being slapped or thrown by objects. Other less frequent forms included being hit with the fist, being grabbed by her arm, being pulled by her hair or being kicked. Being attacked by a weapon was the least commonly reported form of physical violence (1.2%). About 6% of women reported being forced into sexual intercourse by her intimate partner, while being physically or psychologically forced into an undesirable sexual act were reported by 2.2% of women, each.

**Table 1:** Prevalence of exposure to VAW among women aged 18-45 years (Helwan, Cairo, 2018)

**Violence against women among intimate partners**	**Number**	**Percent**
Psychological ^a^ (n=514) ^b^		
Humiliation	186	36.1
Calling names	175	34.1
Threatening to harm	37	7.2
Physical ^a^ (n=514) ^b^		
Pushing	144	28.0
Slapping	97	18.8
Throwing objects at her	89	17.3
Hitting with fist	57	11.1
Grabbing arm	50	9.7
Pulling hair	41	8.0
Kicking	28	5.4
Attempting to suffocate or burn	28	5.4
Attacking with a weapon	6	1.2
Sexual ^a^ (n=496) ^b^		
Forced her into sexual intercourse	29	5.8
Physically forced her to do an undesirable sexual act	11	2.2
Psychologically forced her to do an undesirable sexual act	11	2.2
Non-intimate male partner violence (n=455) ^b^		
Physical harassment	65	14.3
Verbal harassment	390	85.7

^a^ Responses are not mutually exclusive ^b^ Percentages were calculated among ever married women who agreed to answer the question

Women recently exposed to acts of violence by a non-intimate male partner amounted to 9.7%, while 10.9% reported that they were previously exposed to acts of violence by a non-intimate male partner. As regards type of violence, it was physical harassment in 14.3% of cases and verbal harassment in 85.7% of cases.

The frequency of exposure to violence was estimated among women exposed to the different types of violence and who agreed to respond to the questions. Nearly one third of the women indicated that they had been exposed to psychological violence 2-5 times during the last 12 months. Regarding physical violence, nearly one-third of women indicated that this happened once. Another third were exposed to physical violence more than 10 times. Concerning forced sex, half of those reporting having forced sex indicated that this was for 2-5 times. Regarding non-intimate partner violence, nearly half the women who reported exposure to acts of violence from a non-intimate partner were exposed to acts of violence for 2-5 times during the previous 12 months, three quarters were exposed to physical harassment, reported being exposed only once in the previous 12 months, while almost half of those who reported exposure to verbal harassment indicated that this happened more than 10 times during the previous 12 months.

Concerning healthcare-seeking behavior, nearly two thirds (66.2%) of women exposed to IPV reported that they have never asked for healthcare upon exposure to violence, 31.2% reported that they sometimes sought healthcare, while only 2.6% reported that they have always asked for healthcare upon exposure to violence. Reasons for seeking healthcare included the inability to withstand violence (45.8%), fear for her life (27.0%), encouraged by someone else (14.5%) and for the sake of her children (12.5%). The places where healthcare was sought included public and university hospitals (22.8%), private clinics and pharmacies (21.1% each), private hospitals and medical advice from friends or relatives (17.5% each).

The most frequently reported reasons for not seeking healthcare after exposure to violence were feeling danger on themselves (41.4%), and fear for their children (22.7%), and the violence stopped afterward (12.7%). Fear from the husband and from scandal were mentioned by 8.8% and 8.3% respectively. The reason for not seeking healthcare was not having money constituted only 4.8%.

About one third (34.0%) of women had good level of knowledge about VAW, 47.8% had fair knowledge, while 18.3% had poor knowledge. The knowledge score ranged from 3-22 with a mean of 14.61±3.9 points and a median of 15 points. Most women (79.9%) had favorable (did not accept) perspective against VAW, 19.2% had moderate perspective, while only 0.9% had unfavorable (accepted) perspective towards VAW. The perspective score ranged from 5-63 with a mean score of 52.01 ±7.16 points and a median of 54 points.

Table 2 shows that higher percentages of women with fair and good knowledge were exposed to VAW (nearly two thirds each) compared to those with poor knowledge. The mean knowledge score of ever-married women was nearly equal among those exposed and those not exposed to violence with no statistically significant difference. On the other hand, higher proportions of women with unfavorable and moderate perspectives about VAW were exposed to violence compared to those with favorable perspective. The mean perspective score was higher among ever-married women not exposed to violence (52.26 ± 7.15) compared to those exposed (50.43 ±7.23). This difference was statistically significant (*P* =0.001).

**Table 2:** Distribution of women aged 18-45 years according to their level of knowledge and perspectives and their exposure to VAW (Helwan, Cairo, 2018)

**Variables**	**Unexposed to violence** **n=210**		**Exposed to violence** **n=304**		***P*** **value**
	**Number**	**Percent**	**Number**	**Percent**	
Knowledge					0.140
Poor	46	50.0	46	50.0	
Fair	99	38.2	160	61.8	
Good	65	39.9	98	60.1	
Perspective					0.001
Unfavorable	1	16.7	5	83.3	
Moderate	25	24.8	76	75.2	
Favorable	184	45.2	223	54.8	
**Variables**	**Mean**	**SD**	**Mean**	SD	***P*** **value**
Score of knowledge	14.48	3.70	14.70	3.61	0.100
Score of perspective	52.26	7.15	50.43	7.23	0.001

Predictors of knowledge of ever-married women regarding VAW were tested using a logistic regression model. The final model included two variables; occupation and marital status. Neither of them was statistically significant (Table 3). On the other hand, two factors were found to be significantly affecting women’s perspectives (unfavorable and fair=0, favorable=1) about VAW. The first was residence. Women with favorable perspectives were 57% less likely to be from slum areas compared to women with unfavorable and fair perspectives. The second factor was level of education. Women with favorable perspectives were 65% less likely to be illiterate compared to women with unfavorable and fair perspectives. The model correctly classified 79.8% of the cases. Table 3 also shows that residence and level of the perspective were the two factors that significantly affected the exposure to VAW (unexposed=0 and exposed=1). Women exposed to violence were 2.37 times more likely to be from slum areas and were 1.61 times more likely to have unfavorable or fair perspectives about VAW compared to unexposed women. The model correctly classified 61.9% of the cases.

**Table 3:** Predictors of knowledge (model a) and perspectives (model a) of women and predictors of exposure to gender based violence (model c) of ever married women aged 18-45 years (Helwan, Cairo, 2018)

**Independent variables**	**Odds ratio** **(95% CI)**	***P*** **value**	**Sensitivity** **of the model**
**Model a:** Predictors of knowledge regarding violence against women	66.0%
Occupation (housewife)	0.67 (0.40, 1.04)	0.080	
Marital status (single)	1.29 (0.79, 2.11)	0.300	
**Model b:** Predictors of perspectives about violence against women	79.8%
Residence (slum)	0.44 (0.23,0.81)	0.010	
Education (illiterate)	0.35 (0.21, 0.59)	0.001	
**Model c:** predictors of exposure to violence against women	61.9%
Residence (slum)	2.37 (1.49, 3.77)	0.001	
Level of perspectives (unfavorable and fair)	1.61 (1.03, 2.52)	0.040	

Model a: Five variables were used to build the model (age, marital status, level of education, occupation and residence) Model b: Six variables were used to build the model (age, marital status, level of education, occupation, residence and knowledge about VAW) Model c: Seven variables were used to build the model (age, marital status, level of education, occupation, residence, knowledge and perspectives about VAW)

## Discussion

Gender-based violence in Egypt remains an issue of social rights, human rights and of public health importance. Focus on evidence generation for proper mapping and intervention is crucial especially in communities where there is a culture of silence around the topic of domestic violence, which makes the collection of data particularly challenging ^10^.

In the current study, among ever-married women, the rates of exposure to IPV were similar to those reported by other studies from Egypt, including a nation-wide study ^11^ as well as studies in Cairo^12^ and Zagazig ^13^. The study in Minya showed some differences in the ranking of different types of violence where physical and sexual violence were more common than psychological/emotional violence ^14^. These differences could be attributed to difference in definitions and in methods of screening and also differences in beliefs and cultural values.

Exposure to physical and verbal harassment by a non-intimate male partner in the present study was reported by 20.6% of women, while that percentage was expressed in the national GBV costing study in 2015 as an overall of 13% of women aged 18-64 yr were exposed during the past year to any form of violence in public places ^11^. The difference in these figures may be due to differences in the method of measurement wherein our study was a concentration on harassment with obvious distinction between physical and verbal types, while in the national costing study the figure was more rough addressing exposure to any form of violence in public places.

The results of health-seeking behavior of women in response to their exposure to violence were comparable to the results of national GBV costing study ^11^. The health-seeking behavioral motives described in this research were similar to the results reported from different studies, observed that factors strongly associated with a higher likelihood of seeking assistance for physical IPV included frequent abuse or serious aggression resulting in injury ^15-19^. However, women were more open to discussing the reasons of why they are no longer seeking help. These reasons were topped by the women’s perception that they were not in danger (32.7%) followed by their fear that this will affect their children badly (17.9%). Arab culture stresses family loyalty, dignity and reputation (honor). Disclosing domestic violence to a physician may be inappropriate and is perceived as and a form of family deception ^20^.

The present findings showed that only 0.9% of women had unfavorable perspectives towards VAW (accepted violence). In Egypt, the national GBV costing study revealed that two factors affected women’s perspectives, namely: type of residence and level of education of women. Illiterate women and women in rural Upper Egypt showed the highest level of acceptance of violent attitudes/situations ^11^. Women’s education had a safeguarding effect on their acceptance of violence on the condition that the husband had the same schooling level ^21^. This may be because education increases autonomy as well as social and economic empowerment ^22^.

In the present work, the prevalence of exposure to violence was predicted by two factors namely: type of residence and level of perspectives. Both relationships were supported by evidence from two local studies in 2006 and 2009^10,21^.

The current study did not conclude significant associations of exposure to violence with age, education or marital status. However, those were revealed by both the 2014 Egypt Demographic and Health Survey, and the national GBV costing study ^5,11^. This difference can be attributed to the representative nature of the national surveys which is different from the current study setting that included a localized area of slum and non-slum residential.

Limitations of the study: Recall period of 12 months is long and is bound to underreport particularly in issues of verbal violence. Although anonymity was stressed and confidentiality assured, yet it is felt that there is underreporting for fear of affecting their family life. Moreover, the prevailing norms of male privilege among non-educated non-working women in slum areas make them consider some forms of violence as part of life made collection of complete data rather difficult.

## Conclusion

Violence against women, predominantly psychological violence, whether perpetrated by an intimate male partner, a non-intimate male or public space harassment is highly prevalent in parts of the Egyptian community with more concentration in slum areas and among women who accept violent attitudes. Almost all women suffered some kind of violence. They, however, do not seek help most of the time. The results of this study would be used by intervention programs aiming at ending VAW. Programs aiming at addressing VAW using different tools for example education and awareness, physical and mental well-being, counseling and rehabilitation are crucial.

## Acknowledgements

Thanks are due to the Egyptian Red Crescent team of social workers (Mothalath Helwan premises) for their help in data collection.

## Conflict of interest

The authors declare that there was no conflict of interest.

## Funding

None.

## Highlights

- Half of women were exposed to violence from their partners.
- Psychological violence ranked first, followed by physical then sexual violence.
- Verbal and physical harassments were mainly experienced from non-intimate partners.
- Most women exposed to violence did not seek help.
